# Protective effect of *lactobacillus plantarum* on alcoholic liver injury and regulating of keap-Nrf2-ARE signaling pathway in zebrafish larvae

**DOI:** 10.1371/journal.pone.0222339

**Published:** 2019-09-11

**Authors:** Yaping Liu, Xiaoqian Liu, Ying Wang, Cao Yi, Jiahui Tian, Kechun Liu, Jie Chu

**Affiliations:** 1 Biology Institute, Qilu University of Technology (Shandong Academy of Sciences), Ji’nan, Shandong, China; 2 School of Medical Instrument and Food Engineering, University of Shanhai for Science and Technology, Shanghai, China; 3 Department of General Practice of Shandong Provincial Qianfoshan Hospital, Ji’nan, Shandong, China; Nanjing University, CHINA

## Abstract

This research investigated the protective effect of *lactobacillus plantarum* against alcohol-induced liver injury and the regulatory mechanism of Keap-Nrf2-ARE signal pathway in zebrafish. Firstly, a zebrafish alcoholic liver injury model was established using1.0mM of ethanol concentration, then two forms of *lactobacillus plantarum* treatment were designed to perform repair, including a *lactobacillus plantarum* thallus suspension (LPS) and a *lactobacillus plantarum* thallus breaking solution (LPBS). After 24h of alcohol injury, *lactobacillus plantarum* concentrations of 0, 1.0×10^5^, 1.0×10^6^, 1.0×10^7^ and 1.5×10^7^ cfu/mL were added to protect zebrafish larvae. Then with the treatment of *lactobacillus plantarum* after 48h, activities of alanine transaminase (ALT), aspartate transaminase (AST), superoxide dismutase (SOD) and malondialdehyde (MDA) in zebrafish tissue homogenate were respectively determined. Keap-Nrf2-ARE signal pathway related gene expression conditions were also analyzed, including nuclear factor (erythroid-derived 2)-like 2(Nrf2), Kelch like ECH associated protein 1(Keap1), catalase(CAT), hemooxygenase1(HO1) and Glutathione S-Transferase Kappa 1(gstk1). Results showed that: in comparison with the control group, the LPBS with dosage of 1.0×10^7^ cfu/mL remarkably improved the activities of SOD, CAT, HO1and gstk1 in zebrafish larvae liver (*P*<0.05), resulting in significant increase of the protein expression level of Nrf2 (225.78%) and suppression of Keap1 gene expression (73.67%)(*P*<0.01). As confirmed by the results, *lactobacillus plantarum* activated the Keap-Nrf2-ARE signal pathway from the level of transcription, the up-regulation of the expression quantity of Nrf2 protected the organism from oxidative stress and maximally reduced liver injury.

## 1 Introduction

Probiotic is regarded as viable bacteria or micro-ecological regulator [1] deriving from Greek languages of pro and bios, which means being conductive to life [2]. Currently, lactobacillus is mainly taken as the probiotic, in particular, *lactobacillus plantarum* is a common bacterium existing in dairy products, fruits and vegetables as well as meat [3]. It is reported that *lactobacillus plantarum* has the efficacies of resisting against cancer, lowering cholesterol level, boosting immunity and delaying senescence, etc. [4–5]. Due to its characteristic of oxidization resistance *lactobacillus plantarum* has excellent oxide removal capacity [6–7]. Researchers have reported that feeding lactobacillus can lead to changes in the immune state of animals, resulting in the increased host immunity, such as the activity of some immune cells, the contents of antimicrobial substances, immunoglobulin in the blood and the expression of immune factors in immune organs.[8–9], Among them *lactobacillus plantarum* has a certain protective effect for alcoholic liver injury [10–11], and it is already found, by mice research, that *lactobacillus plantarum* may be favorable for prevention and improvement of alcohol-induced fatty degeneration and injury, and meanwhile can restore and improve intestinal balance, so as to lower the serum endotoxin level [12]. However, researches on the mechanism of *lactobacillus plantarum* in repairing alcoholic liver injury are limited, and related research applied to zebrafish alcoholic liver injury protection has not been reported.

Ethanol, a small polar molecule with good water and lipid solubility, has a strong permeability to various biofilms, including the cerebrospinal fluid barrier, and has a significant influence on the function of the nervous system. Animal studies on alcohol injury have shown that ethanol had a damaging effect on motor ability, sensory ability, and even advanced cognitive functions, including learning and memory [13–16]. According to previous studies, ethanol was oxidized to acetaldehyde in the liver, where the acetaldehyde may possibly affect the functions of mitochondria and canaliculi, and formed an acetaldehyde complex by combining with various proteins to become a new antigen, causing injury to the liver cells [17]. In a mouse model, *lactobacillus plantarum* lowered the endotoxin level, down-regulated the expression of TNF-α [18], lowered the levels of ALT, AST and γ-glutamyl transferase (γ-GT) in mice serum, and increased the contents of glutathione (GSH), superoxide dismutase (SOD) and glutathione peroxidase (GSH-PX), leading to the improvement of the oxidization level of the organism, therefore played a role of protecting the liver [19]. Although more researchers are trying to explain the acting mechanism of *lactobacillus plantarum*, few people get to know the pathway regulation, therefore, the exact regulatory mechanism of the *lactobacillus plantarum* to protection of alcoholic liver injury still remains unclear.

Zebrafish, as a novel model animal which has a complete genome sequence of approximately 30,000 genes, processes the homology with human gene exceeding 87% [20]. The cardiovascular system, the immune system and the nervous system of the zebrafish, especially the digestive system such as liver, etc., have a lot in common with corresponding systems of the human [21]. Existing zebrafish hepatotoxicity assessment methods based on gene expression biomarkers and phenotypic analysis have good applicability [22]. These data and technological approaches all support that the zebrafish model has the feasibility for animal hepatotoxicity predication and assessment, due to its unique advantages of short experiment cycle, where the liver of the zebrafish is primarily formed after 48 hpf (hours post fertilization) and fully grows after 72 hpf, [23], therefore, zebrafish is widely used in research as a drug-induced liver injury model. Based on many advantages of zebrafish, this study researched the regulatory mechanism of a *lactobacillus plantarum* extract in a Keap-Nrf2-ARE signal pathway of zebrafish alcoholic injury, assessed the repair efficacy of *lactobacillus plantarum* to the liver by observing the survival conditions, the liver morphological change, the liver tissue pathological assessment and the fluorescent expression condition of liver. We also tested biochemical indicators such as transaminase and dismutase, etc., and performed real-time PCR survey on alcohol-induced hepatotoxicity, thus the present study provided theoretical basis and experimental evidence for the development of the medicinal function of *lactobacillus plantarum*.

## 2 Materials and methods

### 2.1 Zebrafish

The zebrafish AB wild strain and zebrafish liver green fluorescence transgene T_3_ (lfabp:EGFP) was provided by Shandong Academy of Sciences (Jinan, Shandong). The condition of culture was adopted from a zebrafish culture method of Zebrafish Book [24]. Zebrafish were cultured under a condition of ventilation and circulating water. The light cycle was 14 h light / 10 h dark alternation, the water temperature was 28 °C, and the prawns and bait were fed twice a day. The zebrafish culture room was looked after and managed by the staff and workers of Shandong Academy of Sciences.

In the experiment, the suitable paired female and male fish were selected according to a proportion of 2:2 or 1:2 for mating, and the fertilized eggs were collected the next day. The embryos were washed for several times with a 0.5% methyl blue aqueous solution, then the embryos were put into incubators with same illumination periods for light-controlled feeding at 28.5°C, and in this period, dead embryos were removed timely. In this experiment, mature embryo individuals after 72 hpf (hours post fertilization) were selected.

Composition of zebrafish culture water: NaCl 5 mmol/L, KCl 0.17 mmol/L, CaCl_2_ 0.4 mmol/L, MgSO_4_ 0.16 mmol/L, deionized water preparation. After being purified with a purifying device, the water quality reaches: salt rejection 95%-98%, organic rejection >150 MW, bacteria rejection 99%, particle rejection 99%, conductivity <30 μS/cm.

### 2.2 Lactobacillus plantarum

*Lactobacillus plantarum* preserved by Microbiology Laboratory of Biology Institute of Shandong Academy of Sciences was revived and activated, then cultured in an MRS liquid culture medium for two generations for each use. The cultivation conditions are as follows: 37°C, 120 rpm, 24 h cultivation.

Concentrations of *lactobacillus plantarum* to repair zebrafish alcoholic injury used in this study were selected as 1.0×10^5^, 1.0×10^6^, 1.0×10^7^ and 1.5×10^7^ cfu/mL based on previous researches.[25]. Meanwhile, two forms of *lactobacillus plantarum* treatment of *lactobacillus plantarum* suspension (LPS) and *lactobacillus plantarum* breaking solution (LPBS) were applied referring to the method of JIN and Hangeum Kim [26–27].

The LPS was prepared by centrifuging *lactobacillus plantarum* culture for 1 min at 12000 rpm, then discarded the supernatant and washed the pellet of cells using PBS for 3 times, followed by 3 times of washing with culture water. After centrifuging for 1 min, the pellet was collected and resuspended in culture water. The LPBS was prepared by ultrasonication treatment of LPS at 800W for a total period of 15 min with a pause of 8s between every 3s of operation. Then, the suspension was centrifuged at 12000 rpm for 10 min and the supernatant (LPBS) was collected.

### 2.3 Drug treatment

#### 2.3.1 Alcohol treatment

Zebrafish embryos normally developed in 72 hpf were selected and transferred into a six-hole cell culture board (in a 5mL solution with 30 embryos in each hole), and were exposed to ethanol with concentrations of 0.6, 0.8, 1.0, 1.2, 1.5 and 1.8 mM. Zebrafish larvae only treated with fish culture water were used as contrast. The zebrafish embryos after administration were put back to the incubators (28.5°C) to continue culture for 72 h. The exposed solution was changed every 24 hours and the dead embryos were removed to prevent the contamination of other surviving embryos. At least three times of parallel repetition are designed for each concentration group of the abovementioned experiment.

#### 2.3.2 *Lactobacillus plantarum* treatment

After being exposed to alcohol solution for 24 h, zebrafish embryos were washed with culture water. Then, the ethanol solution used to treat zebrafish were replaced by ethanol solution containing LPS or LPBS at the concentrations of 1.0×10^5^, 1.0×10^6^, 1.0×10^7^ and 1.5×10^7^ cfu/mL.before being incubated at 28.5°C for 48 h. Other steps were the same as the alcohol treatment.

### 2.4 Lethal and tratogenesis effects of ethanol to zebrafish

The lethality and teratogenicity of solvent ethanol to the zebrafish after exposure treatment for 24, 48 and 72 hours were observed with a multifunctional microscope (Olympus FSX-100, Tokyo, Japan). The dead zebrafish that did not possess heartbeat was recorded and photographed using the multifunctional microscope (Olympus FSX-100, Tokyo, Japan)with a magnification of 3.2.

### 2.5 Liver morphology observation under fluorescence microscope

The experimental liver green fluorescence transgene T3 (*lfabp*:*EGFP)* strain was collected. Tricaine (0.16%) was used for anesthesia, and the observation was performed using a fluorescence stereoscopic microscope (Olympus SZX16, Tokyo, Japan) with a magnification of 4.2.

### 2.6 Pathological assessment of zebrafish liver tissue

After drug exposure,. Liver tissues of zebrafish were fixed in 4% paraformaldehyde, embedded in paraffin, sliced, dyed with hematoxylin-eosin (HE), and amplified by 30 times under a multifunctional microscope (Olympus FSX-100, Tokyo, Japan) for photographing and observing.

### 2.7 Test on related biochemical indicators of bodies of zebrafish larvae

After exposure treatment for 48 h, zebrafish embryos were all transferred into an EP tube, any remaining solution was removed thoroughly as much as possible. During the test, zebrafish embryos in the EP tube were preserved in ice water. Tissue homogenate was prepared by blending for 3–5 min using a tissue cell breaker (NEXTADVANCE Bullet Blender 24, America). The homogenate was then centrifuged for 10 min at 2500 rpm with a high-speed micro-centrifuge (LG15-W, Beijing) at the temperature of 4°C, and the supernatant was taken. The activitiesof superoxide dismutase (SOD), alanine transaminase (ALT), aspartate transaminase (AST) and trace malondialdehyde (MDA) were determined using kits from Nanjing Jiancheng Bioengineering Institute, Nanjing, China. The absorbance value was measured by a multiscan spectrophotometer (DYNEX Spectra MR, American). Three replicants were conducted for the above experiment.

### 2.8 Real-time quantitative PCR

Thirty zebrafish larvae after exposure for 72h in each group was collected, total RNA of zebrafish was extracted by NanoMag^™^ total RNA isolation from generic samples (Shannuo Scientific Company, Tianjin, China) following the instructions of the kit, and absorbance A260/A280 proportion was recorded with Quickdrop to calculate RNA mass. HiScript II Q RT SuperMix for qPCR(+gDNA wiper) kit (Vazyme, Nanjing, China) was used to perform RNA reverse transcription, and a reverse transcription product was preserved at the temperature of -20°C for later use. A Real-time PCR system consisted of10 μL of 2×SYBR Green qPCR Mix, 1 μL of Forward Primer(10 μM), 1 μL of Reverse Primer(10 μM), 1μL of cDNA and 7 μL of Water (nuclease-free), was uniformly mixed, centrifuged, and distributed to a 8-cascade or 96-hole PCR plate, and 3 PCR parallel reactions were conducted for each gene of each sample. Cycle conditions were as follows one cycle of initial denaturation at 95°C for 5min, followed by 45 cycles of denaturation at 95°C for 10s, annealing at 57°C for 30s, one cycle of the dissociation stage for 10s at 95°C, 60s at 65°C and 1s at 97°C. The amplification curve and melting curve analysis was performed for each reaction. The gene primer was provided by Beijing Jinguoweite Biotechnology LLC, where *β-actin* was a reference gene, and the sequence is shown in Table 1.

**Table 1:** Primers for Real-time PCR.

Gene	Primer orientation	Primer sequence(5’-3’)	Size(bp)
*β-actin*	Forward	ACCCCATTGAGCACGGTATT	135
	Reverse	CTTTGGGATTCAGGGGAGCC	
*Nrf2*	Forward	CTCCAAACCTCCGTTCACCA	133
	Reverse	GTCGTCTACGGGCAGATTGA	
*Keap1*	Forward	GCACTGACCTACACCTTCGC	157
	Reverse	GCCTTGTAGACCTCGCTCTC	
*CAT*	Forward	ACATCACGCGCTACTCCAAA	132
	Reverse	CTGCGAAACCACGAGGATCT	
*HO1*	Forward	GAGTGACGAGCGCCAATAGA	115
	Reverse	AACTCGAATGCTCTGACGGC	
*gstk1*	Forward	TACTTTGGGGTTCCTGTGCG	106
	Reverse	TCTCCTTCTCTGCTACCGCT	

### 2.9 Ethics statement

The study was carried out in strict accordance with the recommendations in the Guide for the Care and Use of Laboratory Animals of the China Academy of Health. The protocol was approved by the Experimental Animal Ethics Committee of the Institute of Biology, Shandong Academy of Sciences (Protocol Number: BISD-20180008). All surgery was performed under sodium pentobarbital anesthesia, and all efforts were made to minimize suffering.

### 2.10 Data processing

Partial results were plotted using Microsoft Excel 2007, and whole data were expressed as mean ± SEM. All experiments were replicated at least three times independently. Statistical significance between different treatments was analyzed by SPSS 19.0 software using single factor analysis of variance (ANOVA). p-values were considered statistically significant if *P<0.05 and statistically highly significant if **P<0.01.

## 3 Result

### 3.1 The toxicity of alcohol to zebrafish larvae

#### 3.1.1 Influence of solvent ethanol to lethality and morphology of zebrafish larvae

**Fig 1:**
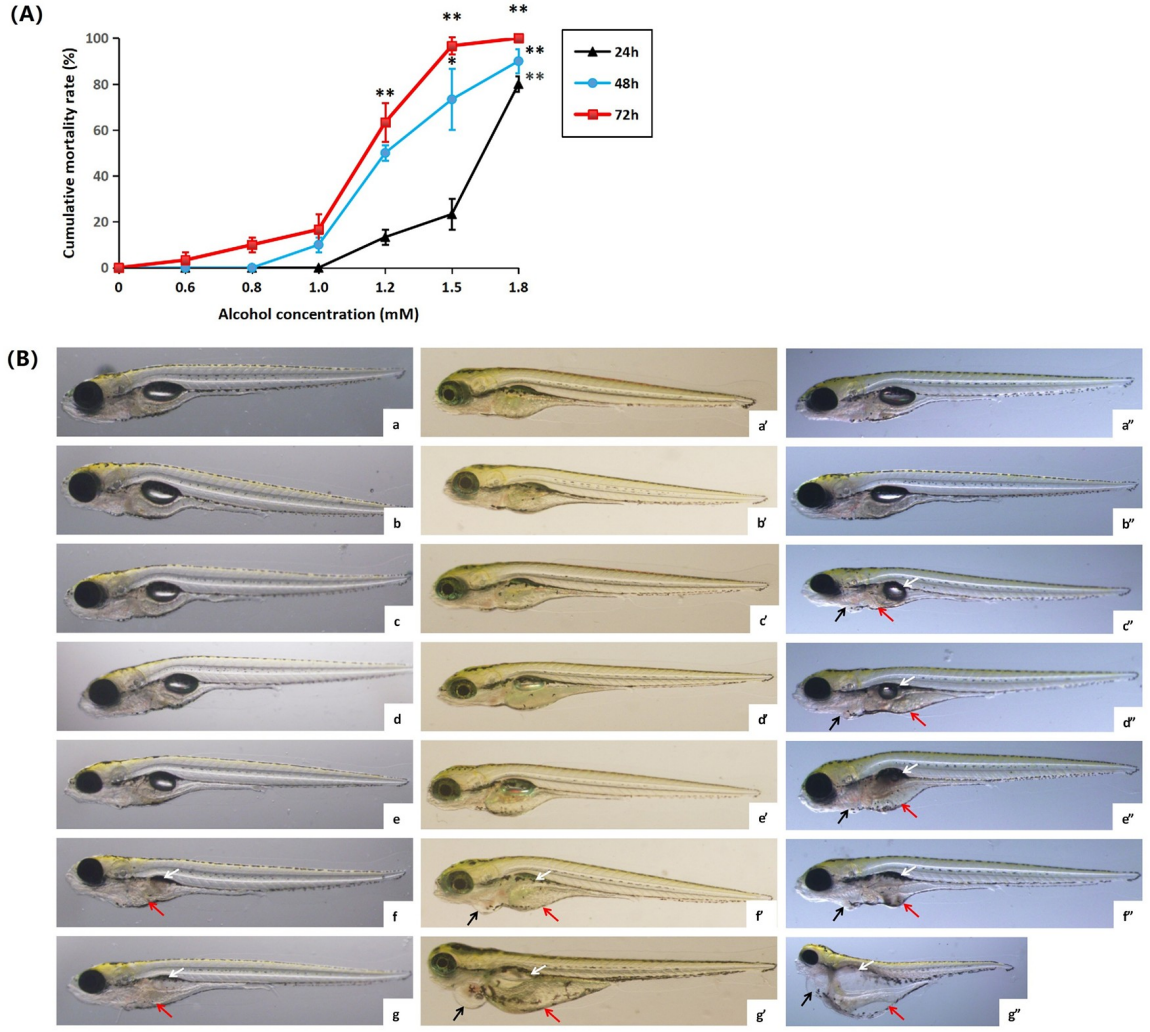
Influence of solvent ethanol to lethality and morphology of zebrafish larvae. (A) Lethality of solvent ethanol to zebrafish after exposure of 24, 48 and 72h at different ethanol concentrations. The experiment was repeated for three times, with 30 zebrafish larvae in each group. Baseline adjusted values (mean±SD) are presented. *P<0.05,**P<0.01 versus control. (B) Influence of solvent ethanol to the morphology of zebrafish larvae after exposure for 24 h (a, b, c, d, e, f and g), 48 h (a’, b’, c’, d’, e’, f’ and g’) and 72 h(a”, b”, c”, d”, e”, f” and g”) at blank (a, a’ and a”) and at the ethanol concentrations of 0.6 mM(b, b’ and b”), 0.8 mM(c, c’ and c”), 1.0 mM(d, d’ and d”), 1.2 mM(e, e’ and e”), 1.5 mM(f, f’ and f”) and 1.8 mM(g, g’ and g”). Swim bladder reduction of zebrafish larvae was pointed out by the white arrow, yolk sac edema was pointed out by the red arrow, and pericardium edema was pointed out by the black arrow.

The lethality of solvent ethanol to zebrafish (Fig 1A) was represented by the lethal dose 50% (LC_50_). Results showed that LC_50_ was 1.71 mM after 24 h, 1.40 mM after 48 h and 1.20 mM after 72 h. After exposure to the ethanol concentration of 1.8 mM, most zebrafish larvae died rapidly after 24h. When the ethanol concentration reduced to 1.0 mM, no obvious influence was induced to the lethality of the zebrafish larvae even after 48 and 72 h.

Meanwhile, the morphological change of the zebrafish larvae under the influence of ethanol was investigated (referring to Fig 1B). The zebrafish embryos exposed in various ethanol concentrations after 72h developed abnormally, showing more evident toxic effect at high concentrations. Compared with the blank group, the liver part after ethanol treatment turned black and lost transparency. According to the result discussed above, the dosage of ethanol and the injury time can affect developmental toxicity change.

#### 3.1.2 Influence of solvent ethanol to liver tissue of zebrafish larvae

**Fig 2:**
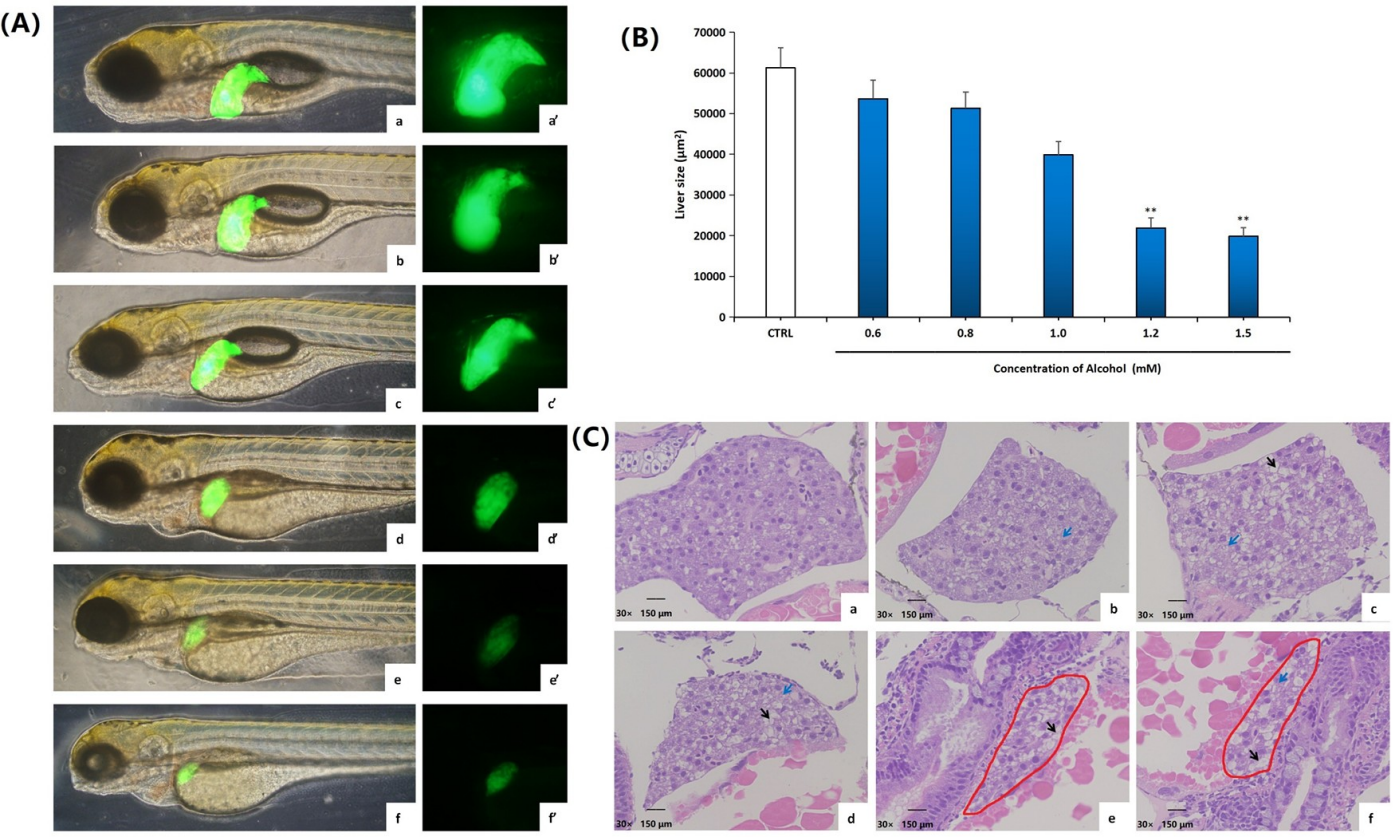
Influence of solvent ethanol to liver tissue of zebrafish larvae. Morphological observation of liver after the liver green fluorescence transgene zebrafish T3 (*lfabp*:*EGFP)* is exposed for 72h at blank (a and a’) and at ethanol concentrations of 0.6 mM(b and b’), 0.8 mM(c and c’), 1.0 mM(d and d’), 1.2 mM(e and e’) and 1.5 mM(f and f’). (A) Test of fluorescence intensity of zebrafish larvae liver in figure 2A with Image-Pro Plus 6.0 software. At least 3 zebrafish were tested in each group of concentration. Baseline adjusted values (mean±SEM) are presented. *P<0.05,**P<0.01 versus control. (B) Pathological observation of zebrafish liver tissue treated in blank (a) and various ethanol concentrations of 0.6 mM (b), 0.8 mM (c), 1.0 mM (d), 1.2 mM (e) and 1.5 mM (f), respectively. The liver part was marked with a red circle. Liver cell fatty degeneration was pointed out by the black arrow. Microvesicular lipid droplets in the liver cell were pointed out by the blue arrow.

In the ethanol treated group, the fluorescence intensity of the zebrafish larvae liver gradually reduced with the increase of alcohol concentration. At the alcohol dosages of 1.2 mM (Fig 2Ae and 2Ae’) and 1.5 mM (Fig 2Af and 2Af’), the fluorescence intensity of the liver almost disappeared, and liver fluorescence area decreased significantly.

With the use of HE dye(referring to Fig 2C), normal liver tissue shape and cell structure were shown in the blank, with complete shape and definite cell boundary, and large vacuoles formed by liver cell fatty degeneration cannot be seen (Fig 2Ca). Whereas in the ethanol treated group, with the increasing ethanol dosages from 0.8 mM to 1.0 mM, 1.2 mM and 1.5 mM, a loose cell structure was observed. Microvesicular lipid droplets and large vacuoles produced by liver cell fatty degeneration can be seen in the liver (Fig 2Cc, 2Cd, 2Ce and 2Cf).

#### 3.1.3 Influence of solvent ethanol to transaminase and oxidative stress of zebrafish larvae

The influence of the alcohol treatment on the contents of ALT, AST, SOD and MDA in the bodies of the zebrafish larvae were determined(referring to Table 2). The increase of alcohol concentration led to the increase of ALT, AST and MDA contents in the bodies of the zebrafish larvae, and the decrease of SOD content. No significant change was observed in alcohol concentration at 0.6 mM.

**Table 2:** Influence on contents of ALT, AST, SOD and MDA in the bodies of the zebrafish larvae after exposure with alcohol of different concentrations for 72h (mean±SD).

Parameter Groups		ALT (U/gprot)	AST (U/gprot)	SOD (U/mgprot)	MDA (nmol/mgprot)
CTRL		4.51±0.37	5.63±0.50	48.82±2.96	0.75±0.05
Alcohol Concentration (mM)	0.6	5.56±0.31	5.92±0.48	44.02±2.65	0.83±0.04
	0.8	7.11±0.59*	7.45±0.54*	34.50±1.40**	1.18±0.09**
	1.0	9.20±0.64**	10.68±0.95**	32.74±1.31**	1.33±0.10**
	1.2	10.12±1.28**	12.4±0.71**	26.33±1.12**	1.42±0.11**
	1.5	10.54±1.23**	13.22±1.84**	23.67±1.32**	1.67±0.23**

*P<0.05,

**P<0.01 versus control.

#### 3.1.4 Establishment of alcohol liver injury model of zebrafish

According to the above results, the death rate of zebrafish larvae was not significant at 1.0 mM alcohol injury for 72 h. Under microscopic examination, the liver of zebrafish showed obvious atrophy and degeneration, and some of the liver cells were loose and vacuolated. Based on the results of the kit test, the level of transaminase increased significantly, while the level of antioxidant enzyme exhibited a significant decrease. Moreover, an obvious oxidative stress reaction was detected. Therefore, 1.0 mM alcohol concentration was used as the model concentration of alcoholic liver injury model of zebrafish larvae.

### 3.2 Protective effect of *Lactobacillus Plantarum* on alcoholic liver injury in zebrafish larvae

#### 3.2.1 Influence of *Lactobacillus Plantarum* to morphology of zebrafish larvae

**Fig 3:**
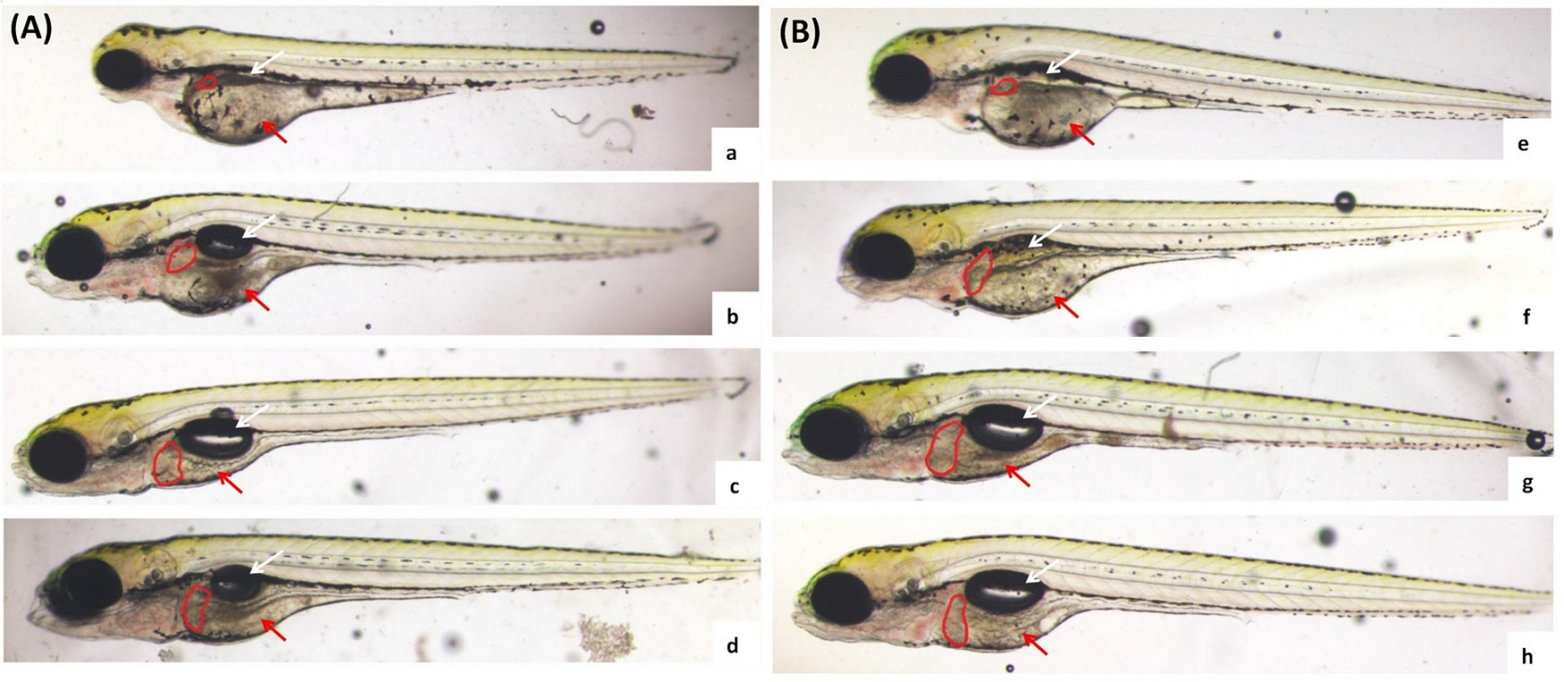
Influence of *lactobacillus plantarum* on morphology of zebrafish larvae. After 24 h of 1.0 mM alcohol injury, replaced ethanol solution with ethanol solution containing LPS with dosages of 1.0×10^5^ cfu/mL (a), 1.0×10^6^ cfu/mL (b), 1.0×10^7^ cfu/mL (c) and 1.5×10^7^ cfu/mL(d) or LPBS with dosages of 1.0×10^5^ cfu/mL (e), 1.0×10^6^ cfu/mL (f), 1.0×10^7^ cfu/mL (g) and 1.5×10^7^ cfu/mL(h) for repair for 48 h, and observe the morphological change of the zebrafish larvae. The liver part was marked with a red circle. Swim bladder reduction of the zebrafish larvae was pointed out by the white arrow, and yolk sac edema was pointed out by the red arrow.

When the *lactobacillus plantarum* concentration increased to 1.0×10^7^ cfu/mL (Fig 3c and 3g) and 1.5×10^7^ cfu/mL (Fig 3d and 3h), the malformation injury was weakened, the swim bladder appeared with luster, and the visible area of the liver was enlarged. Compared between two forms of *lactobacillus plantarum* treatment, LPBS showed better protective effect.

#### 3.2.2 Influence of *Lactobacillus Plantarum* on liver tissue of zebrafish larvae

Compared with the ethanol modeling group with dosage of 1.0 mM, after *lactobacillus plantarum* was added, the fluorescence intensity of the liver increased, and liver injury was relieved to a certain extent. Under the protection of the LPBS with dosage of 1.0×10^7^ cfu/mL, the fluorescence intensity of the zebrafish larvae liver was close to the blank (Fig 2Aa and 2Aa’).

**Fig 4:**
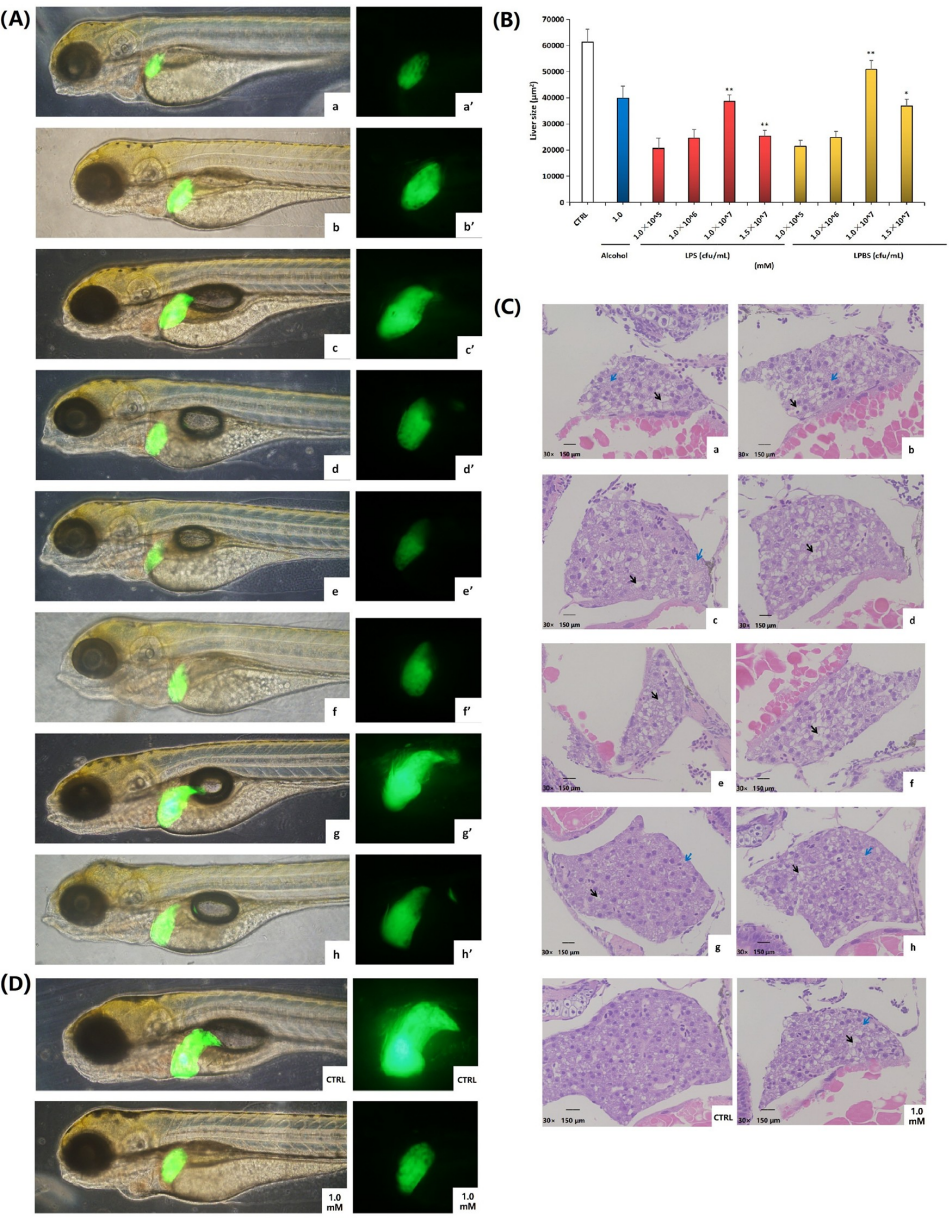
Influence of *lactobacillus plantarum* to liver tissue of zebrafish larvae. Morphological observation of zebrafish liver after the liver green fluorescence transgene T_3_ (*lfabp*:*EGFP)* was exposed for 48 h at LPS concentrations of 1.0×10^5^ cfu/mL (a and a’), 1.0×10^6^ cfu/mL (b and b’), 1.0×10^7^ cfu/mL (c and c’) and 1.5×10^7^ cfu/mL(d and d’), and LPBS concentrations of 1.0×10^5^ cfu/mL (e and e’), 1.0×10^6^ cfu/mL (f and f’), 1.0×10^7^ cfu/mL (g and g’) and 1.5×10^7^ cfu/mL(h and h’). (A) Test of fluorescence intensity of zebrafish larvae liver in figure 4A with Image-Pro Plus 6.0 software. At least 3 zebrafish were tested in each group of concentration. Baseline adjusted values (mean ± SEM) are presented. *P<0.05,**P<0.01 versus control. (B) Pathological observation of zebrafish liver tissue treated with LPS with dosages of 1.0×10^5^ cfu/mL (a), 1.0×10^6^ cfu/mL (b), 1.0×10^7^ cfu/mL (c) and 1.5×10^7^ cfu/mL(d), or LPBS with dosages of 1.0×10^5^ cfu/mL (e), 1.0×10^6^ cfu/mL (f), 1.0×10^7^ cfu/mL (g) and 1.5×10^7^ cfu/mL(h), respectively. Liver cell fatty degeneration was pointed out by the black arrow. Microvesicular lipid droplets in the liver cell were pointed out by the blue arrow. (C) The liver fluorescence intensity and the pathological observation of liver tissue of zebrafish of control group and 1.0 mM alcohol group.

Pathological assessment for zebrafish liver tissue was performed by observing (referring to Fig 4C). With the addition of *lactobacillus plantarum* at 1.0×10^7^ cfu/mL (Fig 4Cc and 4Cg) and 1.5×10^7^ cfu/mL (Fig 4Cd and 4Ch), the shape and cell structure of the liver tissue were observed to be normal with tight cell contact,. In addition, microvesicular lipid droplets and vacuolation were relieved to a certain extent. The LPBS with dosage of 1.0×10^7^ cfu/mL had the optimal protective effect, which was comparable to normal liver cells of the blank group (Fig 2Ca).

#### 3.2.3 Influence of *Lactobacillus Plantarum* to transaminase and oxidative stress of zebrafish larvae

The influence of the alcohol on the ALT, AST, SOD and MDA levels in the bodies of the zebrafish larvae were analyzed (referring to Table 3). With the protection of *lactobacillus plantarum*, change of ALT, AST, SOD and MDA contents were restricted, particularly, ALT and SOD contents in the bodies of the zebrafish larvae were protected by adding the LPBS with dosage of 1.0×10^7^ cfu/mL, showing similar results to blank.

**Table 3:** Influence on contents of ALT, AST, SOD and MDA in the bodies of the zebrafish larvae after exposure with *lactobacillus plantarum* of different concentrations for 48 h (mean±SD).

parameter groups		ALT (U/gprot)	AST (U/gprot)	SOD (U/mgprot)	MDA (nmol/mgprot)
CTRL		4.51±0.37	5.63±0.50	48.82±2.96	0.75±0.05
Alcohol (mM)	1.0	9.20±0.64**	10.68±0.95**	32.74±1.31**	1.33±0.10**
LPTS (cfu/mL)	1.0×10^5^	9.43±0.85**	10.62±1.90**	31.69±0.95**	1.39±0.19**
	1.0×10^6^	7.10±1.07**	9.63±0.74**	36.78±0.77**	1.06±0.12**
	1.0×10^7^	6.29±0.24**	8.13±0.74*	42.14±1.49**	0.99±0.02*
	1.5×10^7^	6.76±0.45**	8.16±1.13*	42.34±2.12**	0.98±0.06*
LPTBS (cfu/mL)	1.0×10^5^	9.23±0.28**	10.01±1.08**	35.91±3.68**	1.39±0.03**
	1.0×10^6^	7.02±0.92**	9.84±1.40**	36.91±3.78**	1.15±0.11**
	1.0×10^7^	5.61±0.12*	7.99±0.55*	43.62±1.51*	0.96±0.01**
	1.5×10^7^	5.85±0.85*	8.40±1.81*	40.70±1.41**	1.09±0.10**

*P<0.05,

**P<0.01 versus control.

### 3.3 Different gene expression analysis

**Fig 5:**
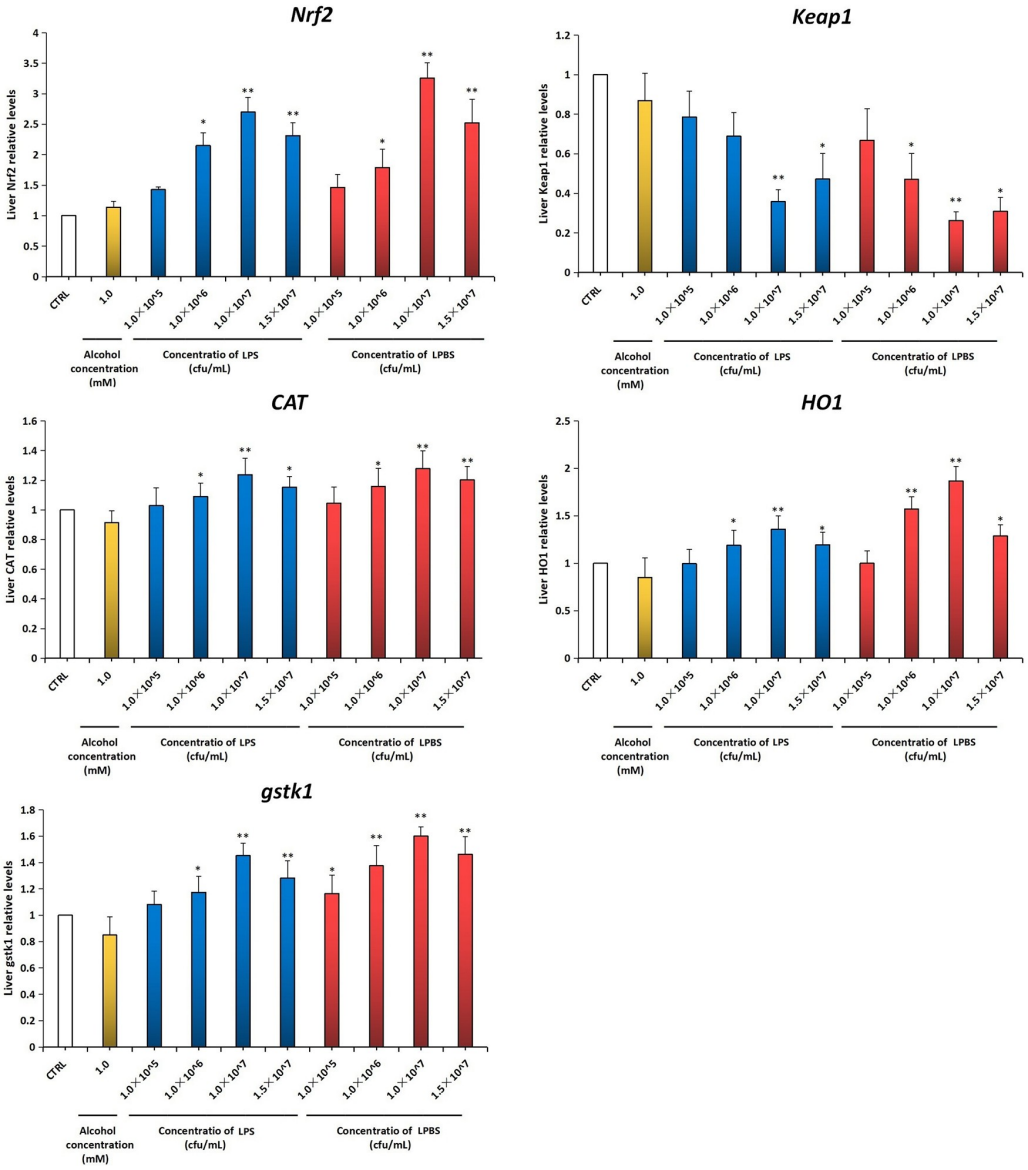
Influence of ethanol injury and *lactobacillus plantarum* repair exposure on expression of related genes of the Keap-Nrf2-ARE signal pathway in the zebrafish liver tissue. Baseline adjusted values (mean ± SEM) were presented. *P<0.05,**P<0.01 versus control.

The Keap-Nrf2-ARE signal pathway is regarded as one of the most important endogenous antioxidant signal pathway in the organism, therefore, we tested several key factors in this pathway. As shown in RT-PCR data (referring to Fig 5), compared with the control group, Nrf2 expression in the zebrafish liver was up-regulated, while Keap1 expression was suppressed after the addition of *lactobacillus plantarum* extract,. Under the protection of the LPBS with the dosage of 1.0×10^7^ cfu/mL, Nrf2 gene expression was remarkably increased by 225.78%, whereas Keap1 gene expression was significantly reduced by 73.67% (*P<0*.*01*). Results also indicated that when exposed to the *lactobacillus plantarum* extract, CAT, HO1 and gstk1 expression were up-regulated at the same time compared to the control group. When exposed to the LPBS with dosage of 1.0×10^7^ cfu/mL, the expression of CAT, HO1 and gstk1 increased by 27.80%, 86.86% and 60.01% (*P<0*.*01*), respectively.

## 4 Discussion

This research first explored the developmental toxicity caused by alcohol of different dosages and treatment time to zebrafish. Along with the increase of alcohol concentration, zebrafish larvae after being exposed for 72 h showed symptoms of pericardium edema, yolk sac edema, swim bladder vanishing, high swimming frequency and spinal curvature, etc. of different degrees at the concentrations above 1.0 mM. By observing under a microscope, the size of the zebrafish larvae liver of 72 hpf reduced with the gradual increase of alcohol dosage. Content of transaminase increased and the content of antioxidant enzyme was lowered. Various data indicated that the liver cells were damaged, which is consistent with the result of a discovery in HE dyeing of zebrafish larvae liver, where the liver suffered from lesion of different degrees. These results were also agreed by previous studies, where spontaneous activities were suppressed due to high-concentration ethanol [28], causing metabolic abnormality and tissue injury [29–30].

Researchers have reported a major mechanism of hepatotoxicity of oxidative stress and apoptosis [31]. ALT and AST were reported to be the commonly used liver function indicators of oxidative stress and apoptosis at present. When lesion occurred to the tissue, the enzyme activity increased [32]. MDA, as an active aldehyde, is one of the main secondary products of lipid peroxidation, thus can be used as the indicator for lipid peroxidation in cell membrane [33]. Another indicator, SOD, can remove superoxide anion free radicals, protect the organism from injury, and plays a crucial role for organism oxidation-antioxidation balance [34]. The result of this research showed that, with the treatment of alcohol, ALT and AST levels increased, MDA level showed a significant increase, while SOD level remarkably reduced (*P*<0.05). Furthermore, oxidative stress was observed to present in the zebrafish liver exposed in alcohol, and CAT, HO1 and gstk1 expression levels were down-regulated, resulting in continuous failure of antioxidant substances formation in the liver and strengthened inflammatory response, finally causing liver apoptosis.

In our research, two different methods were provided to extract *lactobacillus plantarum*, the extract of different concentrations was added to protect zebrafish liver from alcohol injury, and various indicators of the zebrafish were detected again. The result showed that the *lactobacillus plantarum* extract weakened the occurrence of zebrafish larvae malformation, induced the appearance of swim bladder luster, enlarged the liver area, restored the transaminase level, and increased SOD activity in the liver (*P*<0.05), so that liver cells gradually became normal cells. It was also found in this research that, the effect of the LPBS with dosage of 1.0×10^7^ cfu/mL was the best in all treatments. Similar results were reported in rat and human trials recently, where low-dosage lactobacillus remarkably improved activities of SOD, GSH-Px and CAT, and lowered MDA content [35–36]. It has been verified that lactobacillus can be used as a safe and effective biological effect modifier, and exhibit the function of improving oxidative stress, however, the relation between specific acting factors and oxidative stress is still unclear.

**Fig 6:**
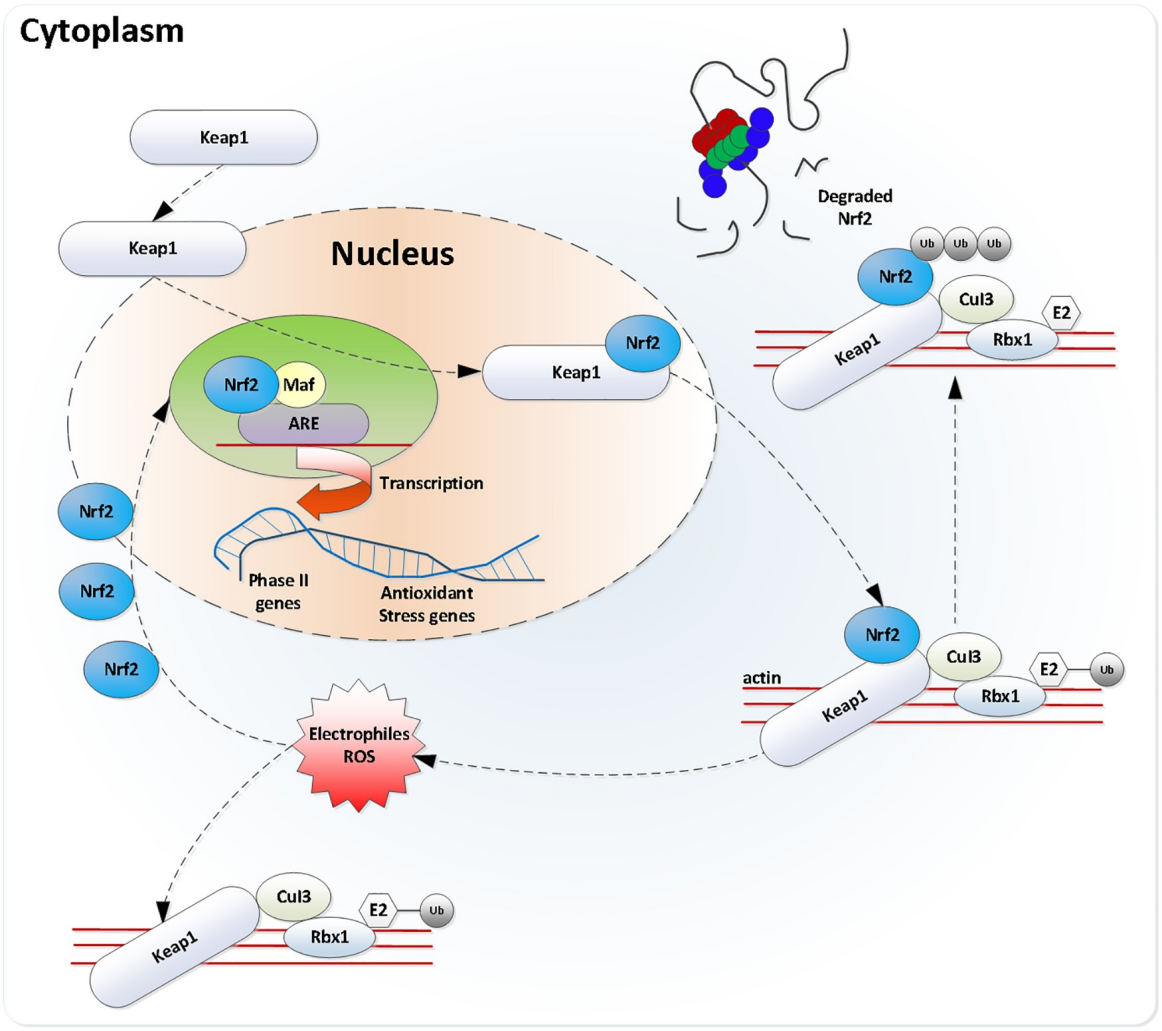
Keap-Nrf2-ARE single pathway [45]. Activated Nrf2 entered the cell nucleus after dissociating from Keap1 suppressing protein, and combined with Maf protein in the nucleus to form a heterodimer and then combined with ARE DNA sequence, therefore, initiated gene transcription controlled by AREThis path is called as Keap-Nrf2-ARE pathway.

According to the above results and numbers of previous studies, the Keap-Nrf2-ARE signal pathway can be recognized as an important endogenous antioxidant signal pathway in the organism in oxidative stress response caused by the organism in coping outside oxidation and chemical substances, etc., [37]. Nrf2, as a key transcription factor, was a switch for activating this signal pathway, and was highly expressed in a detoxication organ, especially the liver [38]. When suffering from oxidation stimulation, a Nrf2-Keap1 compound was dissociated, Nrf2 transferred into the cell nucleus from cytoplasm and was combined with ARE and Maf proteins, therefore, started the transcription of ARE-encoded II phase enzyme genes and certain antioxidant enzyme genes [39–40] (referring to Fig 6). In other studies, exogenous chemicals promoted the activation of Nrf2 in the nucleus. After Nrf2^+/+^ mice were fed with pyrazole, the expression of GST, γ-GCS and HO-1 protein in mouse liver was significantly up-regulated, and the oxidative stress response of mouse liver was significantly alleviated, but the oxidative stress damage of Nrf2^-/-^ mouse liver was significantly aggravated, however, there was no up-regulation of the above protein expression [41–42]; Resveratrol promoted the expression of SOD by activating Nrf2, which reduced the oxidative stress injury of rat liver primary cells stimulated by H_2_O_2_ [43]. More studies have confirmed that exogenous substances can activate the expression of antioxidant genes and play an anti-inflammatory and antioxidant role. The reduction of oxidative stress damage induced by toxic compounds were also confirmed.

One research showed that oxidative stress using relatively high concentration and long time had a function of down-regulating the expression quantity of Nrf2 [44]. As discussed above, the *lactobacillus plantarum* extract was possibly related to the activation of the Keap-Nrf2-ARE signal pathway. In order to further explore the regulatory mechanism of the *lactobacillus plantarum* extract for the zebrafish Keap-Nrf2-ARE signal pathway with alcohol injury, RT-PCR technology was applied in this research to analyze the change of expressions of Nrf2, Keap1, CAT, HO1 and gstk1 genes in the zebrafish liver from the aspect of gene expression. It was found that the *lactobacillus plantarum* extract of different concentrations up-regulated the Nrf2 expression in the zebrafish liver and suppressed the expression of Keap1, and meanwhile up-regulated expressions of CAT, HO1 and gstk1. The research also showed that the *lactobacillus plantarum* extract indeed activated the Keap-Nrf2-ARE signal pathway from the level of transcription, and the up-regulation of the expression quantity of Nrf2 protected the organism from oxidative stress, resulting in the maximum reduction of liver injury.

## 5 Conclusion

This research investigated the influence of the *lactobacillus plantarum* extract on the main antioxidant enzyme of zebrafish larvae with alcohol liver injury, and analyzed a possible regulatory mechanism based on the Keap-Nrf2-ARE signal pathway. It was shown in the research that hepatotoxicity could be caused when the zebrafish larvae was exposed in alcohol, and it was observed that Nrf2 level was lowered in relation to oxidative stress and cell apoptosis. The *lactobacillus plantarum* extract had the functions of chemical prevention and resisting against oxidative stress. It remarkably promoted the activities of SOD, CAT, HO1 and gstk1, increased Nrf2 expression and suppressed Keap1 expression in the aspect of gene transcription, leading to the reduced oxidative injury of alcohol to the organism. This research provided theoretical basis and experimental evidence for development of related functional food or drugs with *lactobacillus plantarum* as an active material.
